# Seuchengeschichte in der deutschsprachigen Urologie

**DOI:** 10.1007/s00120-020-01253-2

**Published:** 2020-07-07

**Authors:** F. H. Moll, T. Halling, M. Griemmert

**Affiliations:** 1 Institut für Geschichte, Theorie und Ethik der Medizin, Centre for Health and Society, Heinrich-Heine-Universität, Düsseldorf, Deutschland; 2 Museum, Bibliothek und Archiv zur Geschichte der Urologie, Deutsche Gesellschaft für Urologie e. V., Düsseldorf, Berlin, Deutschland; 3 Urologischer Arbeitsplatz Krankenhaus Merheim, Kliniken der Stadt Köln GmbH, Neufelder Straße 32, 51067 Köln, Deutschland

**Keywords:** Urologie und Seuchen, Urogenital Tuberkulose, Geschlechtskrankheiten, Seuchengeschichte, Urologiegeschichte, Geschichte der Medizin, Urology and pandemics, Urogenital tuberculosis, Sexually transmitted diseases, History of urology, History of medicine, History of pandemics

## Abstract

Pandemische Erkrankungen haben für viele Gebiete der Medizin Relevanz, von Mikrobiologie über die Epidemiologie bis zur Krankenhausökonomie und die einzelnen medizinischen Teilgebiete. In der sich im 19. Jahrhundert entwickelnden Disziplin Urologie wirkten die pandemische Erkrankung Tuberkulose, sowie die Geschlechtskrankheiten gar fachkonstituierend. Bei Letzteren variierte jedoch häufig die Zuordnung bei der Entwicklung des Krankenhauses im 19. Jahrhundert durch die Differenzierung der „äußeren Klinik“ in die Fächer Chirurgie, Venerodermatologie und Urologie.

## Abstract (en)

Pandemics are relevant for many fields of medicine from microbiology to economics and epidemiology. Many medical specialties which developed during the 19th century, e. g., urology, have had much impact on diagnostics and therapy, such as during the treatment of tuberculosis and sexually transmitted diseases. For some of them, including urology, treatment of, for example, sexually transmitted diseases, was constitutional and differed between countries.

## Einleitung

„Seuchen sind die sozialsten aller Krankheiten. Sie treffen ganze Gesellschaften, schüren kollektive Ängste und verschärfen soziale Spannungen“ konstatierte der Historiker Malte Thiessen in seiner derzeit vielzitierten Analyse der Sozial- und Kulturgeschichte der Seuchen [1]. Die für die Urologie wichtigen und fachkonstituierenden Seuchen wie die Tuberkulose, die Geschlechtskrankheiten (insbesondere Gonorrhö und Syphilis) und im weiteren Sinne Aids [2] sind gleichzeitig in besonderem Maße skandalisierte Erkrankungen [3–5]. Auf diese trifft das wissenschaftshistorische Modell des epidemiologischen Übergangs, der Wechselwirkung zwischen durchschnittlichem Gesundheitszustand der Bevölkerung und sozioökonomischem Wandel – nicht nur durch die häufige Vergesellschaftung von TBC und AIDS – nur bedingt zu ([6–8]; Tab. 1).

**Table 1:** 

1889/1892 Russische Grippe	1 Mio. Todesfälle
1918/19 Spanische Grippe	40 Mio. Todesfälle (ca. 350.000 Deutsches Reich)
1957/58 Asiatische Grippe A/H2 N2	4 Mio. (BRD ca. 29.000 Todesfälle, DDR nicht vorliegend)
1968/69 Hongkong-Grippe A/H3N2	2 Mio. (BRD ca. 30.000 Todesfälle, DDR nicht vorliegend)
1980er HIV/AIDS	>37 Mio. (Deutschland ca. 19.000 Todesfälle 1980–2001)
1991 Cholera	12.000 (nur in Lateinamerika)
2002/03 SARS	774
2003 H5N1 (Vogelgrippe)	450 (250.000–500.000 sterben jährlich an saisonaler Grippe)
2009 Influenza A H1 N1 (Schweinegrippe)	18.449 (253 Deutschland, wahrscheinlich liegt die Zahl um das 10Fache höher)
Malaria 2017	219 Mio. Erkrankungen, Todesfälle 435.000 weltweit (Deutschland 3)
Tuberkulose 2017	10 Mio. Erkrankungen, Todesfälle 1,5 Mio. weltweit (Deutschland ca. 100)
AIDS 2017	1,7 Mio. Erkrankungen, Todesfälle 770.000 (Deutschland ca. 450)

Bis heute stellen pandemische Erkrankungen ein nicht überwundenes Problem von Medizin und Gesellschaft dar, was der derzeitige Ausbruch der COVID-19-Pandemie besonders deutlich macht.

Während zwischen 1980–2000 die Seuchengeschichtsschreibung im Rahmen einer Sozialgeschichte der Medizin ein wichtiges Untersuchungsfeld darstellte, war es in den vergangenen zwei Jahrzehnten vergleichsweise unterrepräsentiert [15, 16]. Gleichwohl bietet die Seuchengeschichte Untersuchungsfelder zu allgemeinen medizinischen, ökonomischen und sozialen Aspekten in der Perspektive einzelner medizinischer Fächer wie der Urologie, Andrologie und Sexualmedizin, um Beziehungen und Verflechtungen sowohl auf globaler als auch auf regionaler Ebene aufzuzeigen [17]. Im Rahmen der aktuellen Pandemie mit dem Corona-Virus SARS-CoV‑2 ist nicht nur die virologische Expertise, sondern auch die historische Einordnung des Geschehens von der Öffentlichkeit nachgefragt [18–23]. Hinzu kommen Online-Ausstellungen wie „COVID-19 & History“ des deutschen medizinhistorischen Museums in Ingolstadt [24–26]. Den urologischen Fachdiskurs selbst dominieren in Bezug auf COVID-19 aktuell Fragen von Priorisierung und Relativierung [27, 28].

## Die historisch-fachliche Rezeption von Seuchen innerhalb der Urologie

Innerhalb der allgemeinen Seuchengeschichtsschreibung waren traditionell insbesondere Pest, Cholera und die Lungentuberkulose und später die „Spanische Grippe“ [29] wichtige Analysefelder [30]. Einschlägige Forschungsfelder sind neben der allgemeinen Einordnung des Seuchengeschehens [31, 32] hier z. B. Fragen zu Stadt-Land-Unterschieden [33–35], der Rolle des Krankenhauses (bzw. seiner Vorläufer) als Behandlungsinstitution, der Beleuchtung gesellschaftlicher und seuchenpolitischer Diskurse [36], wie z. B. der Kritik an seuchenpolizeilichen Maßnahmen [37] oder auch die Frage einer Sinnhaftigkeit retrospektiver Diagnosen [38–40].

Dass die Gefährlichkeit von Tuberkulose und Geschlechtskrankheiten für die Gesellschaft auch im 20. Jahrhundert noch kontrovers diskutiert wurde, lässt sich beispielsweise daran ablesen, dass innerhalb des parlamentarischen Rates 1947/1948 darüber gestritten wurde, ob man bei den Themen Tuberkulose und Geschlechtskrankheiten dem Bund eine Vorranggesetzgebung einräumen sollte [41].

In der Historiographie der Urologie fand eine multiperspektivische Seuchengeschichtsschreibung kaum Beachtung. Dies hat sicherlich eine Ursache darin, dass die häufig aus dem klinischen Umfeld stammenden Forscher [42] andere Forschungsfragen, die eher am Grenzgebiet zur operativen Urologie liegen und lagen, formulierten. Auch werden diese Erkrankungsentitäten in der Regel bis heute interdisziplinär in Klinik und Praxis therapiert und lagen damit häufig weniger im Blickfeld des Interesses einer technikorientieren Historiographie [43]. Den Urologen kam im Verhältnis zu den Venerodermatologen oder den Lungenfachärzten bei der Therapie von Geschlechtskrankheiten (in der Regel Syphilis und Gonorrhö; Ulcus molle sowie Lymphogranuloma inguinale spielten dagegen keine wesentliche Rolle) bei der Urotuberkulose eine besondere Bedeutung zu.

Dies erklärte sich einerseits aus der Patientenpräferenz, sowie therapeutischen Rationalen: Das Aufsuchen eines Urologen, eines „Arztes für Harnkrankheiten“, der bis zum Zweiten Weltkrieg zumeist im stärker anonymen Großstadtbereich angesiedelt war, schien Betroffenen als weniger stigmatisierend: Aus der konsultierten Disziplin konnte nicht sofort auf Erkrankungsentitäten wie Tuberkulose oder auch die Geschlechtskrankheiten geschlossen werden.

Auch bedurften die Organtuberkulose ganz anderer, meist operativer Therapien (ähnlich der gonorrhoischen Urethra Strikturen oder der durch die HIV-Therapie hervorgerufenen Indinavir-Konkremente).

Die Gründung von Fachgesellschaften wie der American Urological Association (AUA) im Jahre 1902 oder der Deutschen Gesellschaft für Urologie e. V. (DGU) 1906/1907 wurden durch Ein- bzw. Ausschluss der Therapie von Geschlechtskrankheiten wesentlich mitgeprägt. Die AUA wurde bewusst unter Ausschluss von Venerodermatologen durch New Yorker Urologen gegründet, wahrscheinlich, um Konkurrenzkonflikten aus dem Wege zu gehen und das Ansehen der neuen Disziplin in den prüden USA zu heben. In den europäischen Großstädten war die Venerologie dagegen ein wichtiges urologisches Betätigungsfeld^1^ [44]. Bei der Gründung der DGU war eine wichtige Untergruppe neben den Endoskopikern die Gruppe der Venerodermatologen um Felix Martin Oberländer (1851–1915, Dresden) oder Arthur Kollmann (1858–1941, Leipzig).

Die Aufklärung der interessierten Öffentlichkeit funktioniert heute v. a. über (digitale) Informationsangebote zu Diagnostik und Therapie von STD/HIV Geschlechtskrankheiten [45–55] oder auch Kampagnen der Deutschen Gesellschaft für Urologie e. V. [56].

In der Historiographie der Urologie finden sich die Erwähnung der pandemischen Erkrankungen wie Tuberkulose oder Geschlechtskrankheiten meist im Zusammenhang mit herausragenden Persönlichkeiten des Faches^2^, in der Angabe einzelner Operationstechniken [57] oder bei der Beschreibung einzelner Therapieprinzipien. Nur in Einzelvorträgen oder einzelnen Handbuchbeiträgen [58] kommt diesen Krankheitsentitäten unter dem Aspekt einer Pandemie‑/Seuchengeschichte eine Reflexionsebene zu, [59–61] obwohl diese in besonderem Maße fachkonstituierend [62] innerhalb der Urologie wirkten.

Andererseits reflektieren medizinhistorische Arbeiten zur Urogenitaltuberkulose [63, 64], zur Tuberkulose i. Allg. [65–67] und zu Geschlechtskrankheiten dies nicht [68–74].

In den Titeln einschlägiger, vielfach aufgelegter Lehrbücher [75, 76] des Fachgebiets Urologie sowie neueren Übersichtsarbeiten [77–84] und Beiträgen in Lehr- und Handbüchern [85–88] sind Geschlechtskrankheiten, Tuberkulose und AIDS bedeutsam.

## Tuberkulose als Prüfstein der operativen Urologie

**Figure 1:**
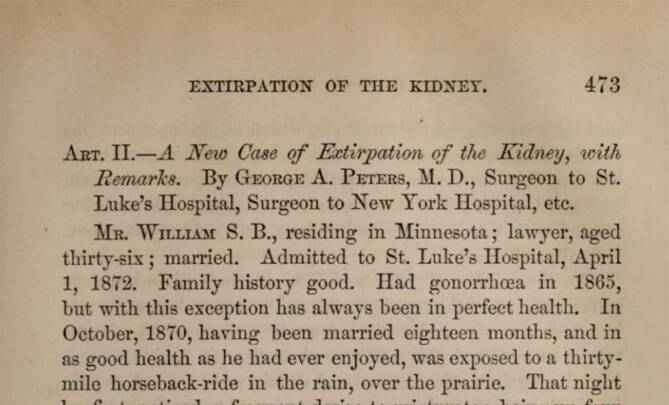

Die Urotuberkulose rückte als Organtuberkulose mit der Möglichkeit, die Nieren operativ behandeln zu können, im letzten Viertel des 19. Jahrhunderts in den Blick der sich entwickelnden medizinischen Spezialdisziplin Urologie, insbesondere durch die Entwicklung operativer Techniken der Nierenchirurgie (Abb. 1).

Ab der Mitte des 19. Jahrhunderts gehörte die „Weiße Pest“ zu den gefürchtetsten Krankheiten, die jährlich Tausende weltweit sterben ließ und Hunderttausende zu Invaliden machte [89]. Die Erforschung der Tuberkulose und die Entwicklung einer potenten Therapie gehörten damit zu den vordringlichsten Anliegen der sich professionalisierenden Medizin. An diesem Prozess waren Forscher(teams) verschiedener Nationen beteiligt:

George Absalom Peters (1825–1894, St. Luke’s and New York Hospital in New York) konnte 1872 erstmals – eher zufälligerweise – eine tuberkulöse Niere entfernen [90, 91]. Die Beschreibung des Mycobacterium tuberculosis 1882 durch Robert Koch (1843–1919) [92] war neben der Klärung der Ätiologie der Organtuberkulose der Nieren (Felix Guyon 1831–1920) – Aszensionstheorie ab 1890 (Keimverschleppung über die Blase oder den Harnleiter bei Untersuchungen) vs. Friedrich Pels-Leusden (1866–1944) – hämtogene Aussaat von einem Herd in der Lunge oder im Darm, ab 1905 [93] vor Einführung einer antibiotischen Therapie Ende der 1940er-Jahre ein wichtiger Schritt zur Klärung von Pathogenese und definitiver Therapie [94]. In der Regel stand die operative Machbarkeit neben der Beschreibung der klinisch oft variierenden Symptomatik und der Diagnostik (Zystoskopie, retrograde Pyelographie, Ausscheidungsurologie) im Vordergrund urologischer Publikationen in Lehrbüchern oder Operationslehren.

Initial waren Nephrektomien bei Tuberkulose umstritten. Galten sie den deutschsprachigen Lehrbüchern der 1890er-Jahren noch als kontrainduziert, teilten die späteren Auflagen des frühen 20. Jahrhunderts diese Einschätzung nicht mehr.

Die operative Therapie der Nierentuberkulose gehörte schon zu Beginn des 20. Jahrhunderts zu den wichtigen Aufgaben im Bereich der Nierenchirurgie innerhalb der Urologie. Viktor Schmieden (1874–1945), später Ordinarius in Halle und Frankfurt, stellte 1902 in Bonn aus der Literatur sämtliche Fälle von nierenchirurgischen Eingriffen zusammen. Bei 1118 aufgeführten Nephrektomien lag die Todesrate insgesamt bei 301 Patienten. Er berichte über 329 Fälle von Operationen bei Karzinomen und 201 Fällen bei Tuberkulose [95].

**Figure 2:**
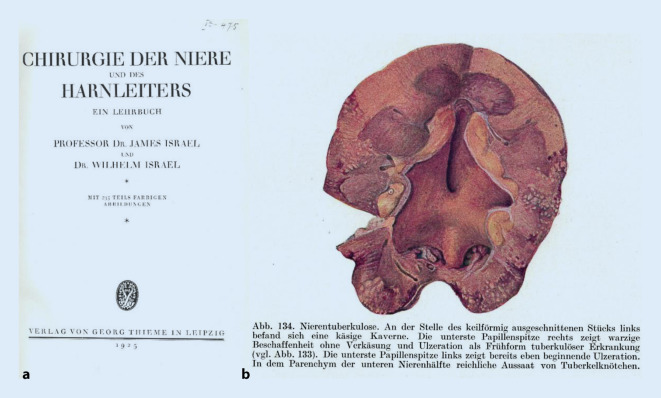

James Israel (1848–1926; [96]), wohl der wichtigste Protagonist der Nierenchirurgie im deutschsprachigen Raum an der Wende zum 20. Jahrhundert, widmete in seinem Lehrbuch 1925 sowohl der Behandlung der Nierentuberkulose wie der Nierensyphilis eigene Kapitel [97]. Dies zeigt deutlich die historische Bedeutung dieser pandemischen urologischen Erkrankungen für das Fachgebiet auf. Hier lässt sich die Wissens- und Wissenschaftsentwicklung im ersten Viertel des 20. Jahrhunderts sowohl in den pointierten Stellungnahmen des Autors, wie in der Literaturauswahl sowohl in Diagnostik wie Therapie gut zurückverfolgen. Seine Forschungsergebnisse argumentierten in der Frage aszendierender bzw. deszendierender Diagnostik gegen den ebenfalls prominenten Fachvertreter Leopold Casper (1859–1959; Abb. 2).

**Figure 3:**
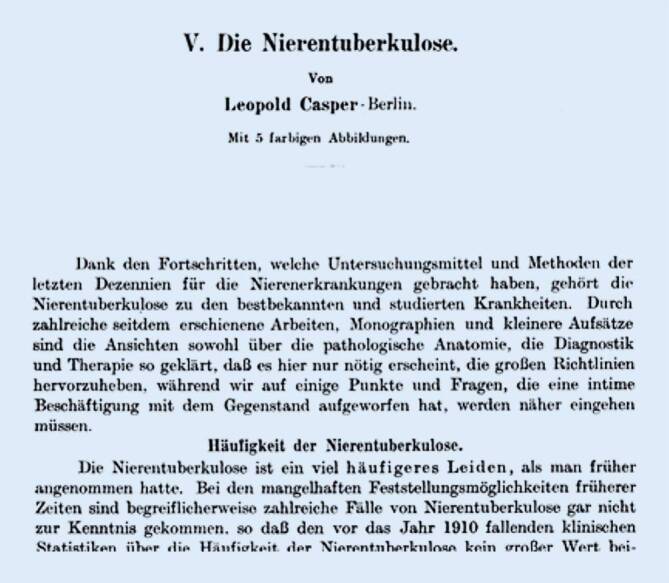

Casper konnte diese einzelnen Entwicklungen 1900/1905 und 1920 in richtungsweisenden Übersichtsaufsätzen zur urologischen Organtuberkulose ebenfalls gut herausstellen [98–100], wobei er auf die Wertigkeit der retrograden Pyelographie und sein Theoriegebäude der „Funktionellen Nierendiagnostik“ rekurrierte (Abb. 3).

Weitere wichtige Akteure und Forschungsschritte des 20. Jahrhunderts seien im Folgenden kurz genannt:

- Die Diskussion um die Bedeutung der Zystoskopie zur Diagnosesicherung führte auch Walter Stoeckel (1871–1961), Gründungsmitglied der DGU für das Grenzgebiet der Urogynäkologie [101].
- Hans Wildbolz (1873–1940; [102]), Bern, fasste den Themenkomplex für das Handbuch der Urologie von Voelcker und Lichtenberg mit seinem Assistenten Walthard in den 1920er-Jahren zusammen [103].
- Der Schwedische Urologe Einar Ljungreen (1896–1986) beschrieb nach dem Zweiten Weltkrieg für die tuberkulostatische Ära diesen Themenkomplex in einem Handbuchbeitrag [104] für den europäischen Raum.
- Hans Boeminghaus (1893–1979) stellte dem Erkrankungskomplex in seinem nach Organeingriffen gegliederten, mehrfach aufgelegten Handbuch „Urologie Operative Therapie Indikation und Klinik“, das mehrere Auflagen erlebte, ebenfalls ein eigenes Kapitel zur Verfügung [105].
- Ferdinand May (1898–1974) konnte 1960 die langfristigen Operationsresultate „state oft he art“ in einem Übersichtartikel für die Zeitschrift *Urologia internationalis* übersichtlich zusammenfassen [106].

Der Vergleich von verschiedenen Auflagen auch in der Urologie verbreiteter Operationslehren lässt den Wandel zu organerhaltenden Eingriffen neben dem Wechsel der Autorenschaft von ausschließlich Chirurgen hin zu Urologen gut erkennen. In der ersten Auflage der Operationslehre Martin Kirschners (1879–1942) von 1937 finden die Blasenerweiterungsplastiken keine Erwähnung und das Stichwort „Tuberkulose“ wird im Sachverzeichnis nicht aufgeführt. Demgegenüber kommt dem Kapitel zur Urotuberkulose bereits in der 2. Auflage von 1961, die von dem Mannheimer Urologen Leonhard Lurz (1895–1977) zusammen mit seinem Sohn Hans Lurz (1922–1995) verantwortet wird, ein wichtiger Stellenwert bei den Eingriffen an der Niere, am Harnleiter oder an der Blase zu [107, 108].

Die Fortschritte der antituberkulostatischen Therapie nach dem Zweiten Weltkrieg ab den 1950er-Jahren lassen sich auch in den verschiedenen Auflagen der chirurgischen Operationslehre von Bier-Braun-Kümmell herausarbeiten, die ebenfalls über einen längeren Zeitraum erschien. Während in der Auflage von 1920 zu Indikation der Nephrektomie die Nierentuberkulose gerechnet wird [109] und Blasenerweiterungsoperationen bei tuberkulösen Schrumpfblasen noch keine Erwähnung finden, ist in der 7. Auflage von 1957 der Nierenteilentfernung bei Tuberkulose ein Absatz gewidmet, der besonders auf die tuberkulostatische Vorbehandlung mit Antibiotika eingeht [110]. In der folgenden Auflage von 1977 stellt der Autor dann fest:

… hat man die früher häufigen Teilresektionen bei Tuberkulose wegen der guten Erfolge der tuberkulostatischen Therapie weitgehend eingestellt … [111]

In dieser Auflage ist, wie in den vorausgegangenen Auflagen, kein Hinweis zu Blasenerweiterungsplastiken bei tuberkulöser Schrumpfblase enthalten, obwohl der Autor des Kapitels zu den Blaseneingriffen, der Urologe Hans Dettmar (1918 –1995), Düsseldorf, die Originalpublikation seines Essener Kollegen Karl Scheele (1884–1966) zitiert [112–114]. Hier könnte der Grund darin liegen, dass sich diese Operationslehre in erster Linie an Chirurgen wandte. Zu dem genannten Zeitpunkt 1977 lagen für den nun auch universitär etablierten Fachbereich Urologie eigenständige Operationslehren vor, die die Nierentuberkulose und Blasenerweiterungsplastiken in eigenständigen Kapiteln ausführlich behandelten und die Zahl der Tuberkuloseerkrankten in der Urologie nahmen bereits wieder ab [115, 116].

Somit sind die in verschiedener Auflage erscheinenden Operationslehren ein guter Indikator des Therapiewandels im Fachgebiet.

Noch in den 1950er- und 1960er-Jahren waren auch in chirurgischen Fachzeitschriften im In- und Ausland Beiträge zur Chirurgie der Tuberkulose an den Harnorganen häufiger zu finden [117–122].

**Figure 4:**
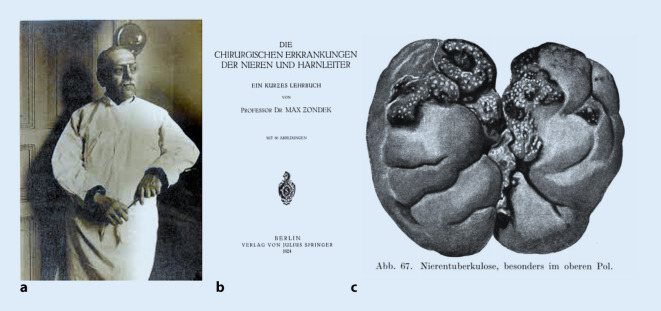

Das Gebiet der Urotuberkulose zeigt, dass sich die meisten prominenten Fachvertreter der Urologie mit dem Themengebiet der Organtuberkulosen ausführlich beschäftigten (Abb. 4). Neben den fachspezifischen Publikationsorganen wie *Zeitschrift für Urologie* oder *Zeitschrift für urologische Chirurgie* nutzten sie auch dem Erkrankungskomplex eigens gewidmete Fachzeitschriften wie die *Beiträge zur Tuberkuloseforschung* (zwischen den 1930–1950er Jahren) oder allgemeinmedizinische Organe wie die *Wochenschriften* (s. oben) neben den chirurgischen Fachzeitschriften zur fachpublikumswirksamen Kommunikation ihrer Forschungsergebnisse in die Chirurgie oder die Allgemeinmedizin.

## Geschlechtskrankheiten als fachkonstituierende Erkrankungen

Die Therapie der Geschlechtskrankheiten, insbesondere die Behandlung gonorrhoischer Harnröhrenstrikturen, gehörte mit der Entwicklung der naturwissenschaftlichen Urologie ab dem 19. Jahrhundert zu den grundlegenden Optionen im Fach [123].

Geschlechtskrankheiten werden als „sozial konstruiert“ bezeichnet, da neben den jeweiligen medizinischen Wissenssystemen wichtige gesellschaftliche Legitimationssysteme sowie religiöse Vorstellungen einflossen, die neue Verpflichtungen und Abhängigkeiten der Patienten hervorriefen [124]. Die Geschlechtskrankheiten erregten und erregen in besonderem Maße Ängste, Scham oder Peinlichkeiten und bildeten den Kristallisationspunkt für „Rituale“, die den Umgang mit den Erkrankten regulierten [125, 126].

Vor der Differenzierung zwischen Syphilis und Gonorrhö [127] 1837 durch Philippe Ricord (1800–1889) und dem Nachweis des Gonokokkus 1879 durch Albert Neisser (1855–1915; [128]) war eine genaue Zuordnung der klinischen Erscheinungen zu den jeweiligen Geschlechtskrankheiten unmöglich:

Seit der frühen Neuzeit wurden sämtliche vesikale Harnabflussstörungen, sei es prostatisch oder strikturbedingt, unter dem Oberbegriff „Karnositäten“ subsummiert und es stand, wahrscheinlich durch Ambroise Paré (1510–1590) inauguriert, nur eine Therapie mit Caustika (Alaun, Schwefelarsenik, Höllenstein/Argentum nitricum) zur Verfügung, das an einer Katheterspitze appliziert wurde [129]. Seit dieser Zeit wurde auch der „Tripper“ („chaude pisse“) als Ursache für Harnröhrenstrikturen angesehen [130].

Auch die Therapie der Bubonen durch Inzision war eine allgemeine wundärztliche Therapiemethode, die schon die handwerklich ausgebildeten Steinschneider oder Barbierchirurgen ausführten [131].

Zu den frühen Promotoren einer verbesserten Operationstechnik mit Angabe eigener Instrumente gehörten in Paris Jean Civiale (1792–1867, Hôpital Necker; [132]) oder Jacques Gilles (Thomas) Maisonneuve (1809–1897, Hôpital Bicêtre, Cochin, Pitié, ab 1862 Hôtel-Dieu; [133, 134]), sowie Fessenden Nott Otis (1825–1900) in den USA [135].

Bereits um 1900 erschienen umfangreiche Lehr- und Handbücher zur urethralen Therapie bei Geschlechtskrankheiten aus der Feder von Urologen wie Felix Martin Oberländer (1851–1915, Dresden; [136]) oder Hans und Erich Wossidlo (1854–1918/1882–1931, Berlin; [137, 138]). Arthur Kollmann (Leipzig) hatte bereits 1888 eine wichtige Arbeit zur Syphilistherapie von Johnathan Hutchinson (1828–1913) übersetzt [139].

**Figure 5:**
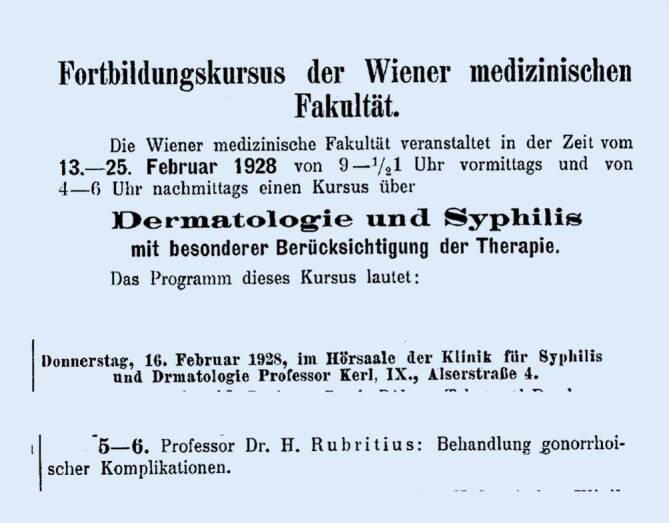

Wilhelm Israel (1881–1959, London), der Sohn James Israels, verfasste für das Handbuch von Voelcker und von Lichtenberg (Julius Springer, Berlin) einen Übersichtsbeitrag zur Syphilis innerhalb der Urologie [140]. Der Schriftführer (bis 1933) der alten DGfU, Arthur Lewin (1866–1939), war mit der Abfassung des grundlegenden Beitrags zu den entzündlichen Erkrankungen der Harnröhre [141] betraut worden, der naturgemäß neben der Urethroskopie den wichtigen Aspekt der konservativen und operativen Gonorrhötherapie enthielt. Urologen wie Rudolf Chwalla (1900–1966), Wiener Allgemeines Krankenhaus, publizierten Fragenstellungen zur Gonorrhö und nutze neben der *Zeitschrift für Urologie* [142] besonders dermatologischen Zeitschriften als Forum zur Präsentation wissenschaftlicher Ergebnisse [143], genau wie Hans Rubritius (1876–1943), Wiener Allgemeine Poliklinik. Dieser veröffentlichte erfolgreich in allgemeinen Publikationsorganen und hielt Vorträge in Wien bei venerodermatologischen Fortbildungen ([144]; Abb. 5).

**Figure 6:**
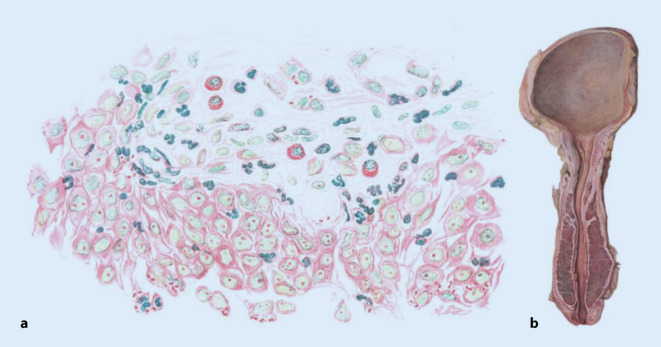

Alexander von Lichtenberg (1880–1949; [145]) und Hans Boeminghaus [146] publizierten ebenso wie Arthur Kollmann [147] wichtige Handbuchartikel in den 1920er-Jahren und erhielten so zur Darstellung der „seuchenhygienischen“ urologischen Aspekte prominenten Publikationsraum, was die gefestigte Etablierung des Therapiefelds innerhalb der deutschsprachigen Urologie durch die Auswahl arrivierter jüngerer und älterer Autoren durch die Handbuchherausgeber gut unterstreicht und hervorhebt. In der *Zeitschrift für Urologie* erschienen in den 1920er-Jahren vielfältige Einzeluntersuchungen zu Fragen von Geschlechtskrankheiten ([148, 149]; Abb. 6).

Da die grundlegenden operativen Therapieprinzipien der seltenen renalen Erscheinungen bei Syphilis [150] und häufigen bei Tuberkulose („Gummen“, Abszesse, Kavernen) ähnlich waren, entwickelte sich in Bereich der Nierenchirurgie hier kein differenziertes technisches Handlungsfeld.

## AIDS

Die enge Verbindung von Urologie und dem HIV-Erkrankungskomplex ist vor wenigen Monaten mit der Erlaubnis zur Verschreibung der PrEP (Präexpositionsprophylaxe) wieder sichtbar geworden [151, 152].

Das Problem der Indinavir-Steine (Crixivan®; [153, 154]), die durch eine HIV-Therapie entstehen, ist Teil der 2018 neu überarbeiteten Leitlinie Urolithiasis des Arbeitskreis Harnsteine der Akademie der Deutschen Urologen der Deutsche Gesellschaft für Urologie e. V. DGU [155]. Die Erkrankung spielt ebenfalls bei facheinschlägigen Hygienerichtlinien und Instrumentenaufbereitung seit den 1980er-Jahren eine wichtige Rolle [156, 157].

## Urologie und soziale Konstruktion von Pandemien

**Figure 7:**
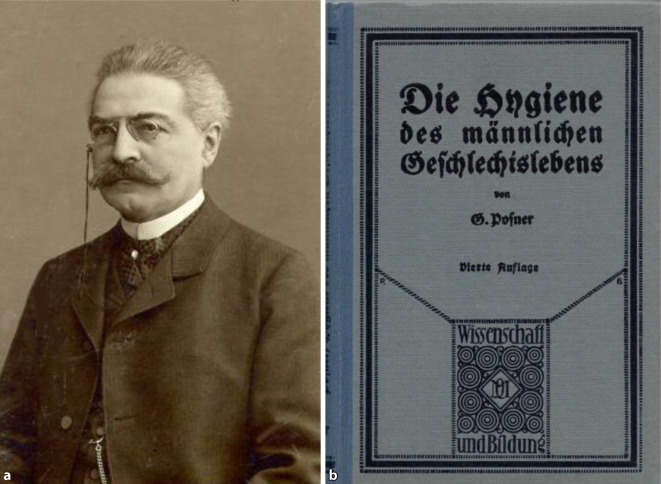

Carl Posner (1854–1928; [158, 159]), Urologe und Sexualmediziner ist ein wichtiger Protagonist, an dessen Beispiel sich die vermehrte Beschäftigung mit Fragen zu Sexualität und Gesellschaft am Beginn des 20. Jahrhunderts innerhalb der deutschen Urologie und Medizin demonstrieren lässt: Posner publizierte neben fachwissenschaftlichen Artikeln [160] eine Aufklärungsbroschüre „Hygiene des männlichen Geschlechtslebens“ [161] bei dem renommierten Verlag Quelle und Meyer in Leipzig, die mehrfache Auflagen erlebte. Hier nahm Posner zum Problem von Prostitution, Meldepflicht von Geschlechtskrankheiten und „Gebrauch von Schutzmitteln“ dezidiert in einem gesonderten Kapitel Stellung, wobei er die Auffassung der 1902 gegründeten „Deutschen Gesellschaft zur Bekämpfung der Geschlechtskrankheiten“ (DGBG) referierte und einem allgemeinen Publikum gegenüber anschaulich darlegte (Abb. 7).

Die DGBG kann sicherlich als die bedeutende Interessengruppe verstanden werden, die sich für eine Gesundheitsreform im Bereich der Sexualpolitik ab der Wende zum 20. Jahrhundert einsetze [162]. Bis in die Weimarer Republik wurden Geschlechtskrankheiten als besondere Bedrohung des „Volkskörpers“ von innen heraus angesehen und zwar sowohl durch die Geschlechtskrankheiten selbst als auch durch deren angenommene Konsequenz, den Rückgang der Geburtenziffer. Hierdurch schienen das Arbeitskräftepotential und die Wehrkraft bedroht. Bis in die Weimarer Republik war ein wichtiger Grundsatz einer Hygienepolitik als Kampf gegen die Geschlechtskrankheiten, die Sexualität diskursiv an die bürgerliche Ehe zu binden, indem diese ausschließlich im Dienste der Fortpflanzung stehen sollte [163].

Hans Haustein (1894–1933 Berlin), sozialistisch orientierter Venerologe Urologe/Sexualmediziner^3^, sozialhygienischer Berater des Deutschen Roten Kreuzes^4^ sowie Inhaber einer Modepraxis in Berlin und Konkurrent von Gottfried Benn (1886–1956; [164]), weiterhin Medizinhistoriker der Abteilung für Genetik des Kaiser-Wilhelm-Instituts für Hirnforschung in Berlin, bearbeitete für Adolf Gottsteins (1857–1941) „Handbuch der sozialen Hygiene“ den Part „Geschlechtskrankheiten“ [165], sowie für das „Handbuch der Haut und Geschlechtskrankheiten“ von Josef Jodassohn (1863–1936) das Kapitel „Statistik der Geschlechtskrankheiten“ [166]. Eine „sozialhygienische“ bzw. auch „rassenhygienische“ wissenschaftliche Betätigung war für niedergelassene Urologen zu dieser Zeit nicht ungewöhnlich, wie das Beispiel von Wilhelm Schallmayer (1857–1919), Düsseldorf oder Benno Chajes (1880–1938 Ascona), zeigt [167, 168].

Gerade die statistische Aufarbeitung war eine wesentliche Grundlage des sich ebenfalls entwickelnden Gebiets der Sozialhygiene [169]. Auf medizinhistorischem Gebiet setzte sich Hans Haustein mit der Geschichte von Syphilis und Tripper auseinander [170]. Hier war er ein Verfechter der sog. Neuwelttheorie der Syphilisentstehung, die den Ursprung der Erkrankung in Amerika und eine Einschleppung durch Christoph Kolumbus vertritt [171].

Die Zusammenfassung der beiden Erkrankungsentitäten Tuberkulose und Geschlechtskrankheiten in einem sozialhygienischen großen Handbuch, welches Themenstellungen des neuen Forschungsfeldes breit ausleuchten und den Wissenskanon der Zeit zusammenfasste, unterstreicht den besonderen Stellenwert, den diese beiden Erkrankungen für die Urologie im Besonderen und die Medizin im Allgemeinen besaßen.

**Figure 8:**
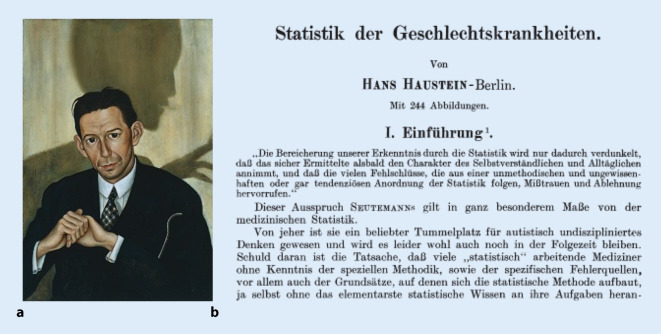

Im genannten „Handbuch der sozialen Hygiene“ hatte sich der Sozialhygieniker Ludwig Teleky (1872–1957), Bruder der Gynäkourologin Dora Teleky (1879–1963), [172] selber das wichtige Kapitel „Tuberkulose“ vorbehalten (Abb. 8).

## Fazit für die Praxis

- Die Therapie von seuchenartigen, pandemischen Erkrankungen wie Tuberkulose oder Geschlechtskrankheiten gehörte und gehört zu den fachkonstituierenden Elementen der sich entwickelnden naturwissenschaftlichen Urologie ab dem Ende des 19. Jahrhunderts und damit auch die Auseinandersetzung mit gesellschaftspolitischen Fragen. Fast alle wichtigen zeitgenössischen Fachvertreter publizierten zu diesen Forschungsfragen, wobei sie neben den fachspezifischen Publikationsorganen auch die monothematisch diesen Erkrankung gewidmeten, sowie die allgemeinmedizinischen Zeitschriften frequentierten. Diese Aktivitäten leisteten einen wesentlichen und wichtigen Beitrag zur Sichtbarmachung der neuen Fachdisziplin Urologie.
- Urologen/Sexualmediziner begriffen die soziale Konstruktion dieser Erkrankungen, da sie sich wie beispielsweise Carl Posner oder Hans Haustein den gesellschaftlichen Implikationen stellten entweder durch volkstümliche Aufklärungsschriften oder „sozialhygienische Publikationen“ und dieses Feld nicht allein Dermatologen oder Sozialhygienikern überließen.
- Bis heute sind diese Erkrankungsentitäten wichtige Bestandteile von Lehr- und Handbüchern oder von Fortbildungsliteratur oder Imagekampagnen.
- Die seuchenhistorische Forschung – auch innerhalb der Urologie – kann den Blick schärfen für die oft unerwünschte und zudem meist die Effizienz beeinträchtigende Indienstnahme von Gesundheitsfragen zur Erreichung gesellschaftspolitischer Ziele.

## Footnotes

1 „… Gonorrheal urethritis remained within the purview of the specialty, but dermatology and syphilology were excluded“, Jones L, Peters P C, Husser W. C. (2002) The American Urological Association Centennial History 1902–2002 American Urol. Ass, Baltimore, S 6; Hansson, N., Halling, T., Moll, F. (2019) Medizin und Literatur: „Nobelpreis. Bitte keine Witze!“ Gottfried Benn und seine Nominierungen für den Literaturnobelpreis Urologe· 58 1481–1488 10.1007/s00120-019-0983-5

2 In der Erinnerungskultur der deutschsprachigen Urologie wird die Therapie der Tuberkulose besonders mit folgenden wissenschaftlichen Werken in Zusammenhang gebracht: James Israel (1848–1926) Die Endresultate der Nephrektomien bei Nierentuberkulose. Fol. Urol. 6 257 (1911). Ferdinand May (1898–1978) Wandlungen in der Behandlung der Urogenitaltuberkulose DMW 80 (14) 479–483 (1955) 10.1055/s-0028-1116455, Thorkild Rovsing (1862–1927) Die Urogenitaltuberkulose. Z Urol 3 315–319 (1909). Einar Ljunggren (1896–1986) Urogenital tuberculosis Inflammation II Handbuch der Urologie S 1–221 (1959) DOI 10.1007/978-3-662-38400-8_1, Hans Wildbolz (1873–1940) Die Tuberkulose der Harnorgane. Marold (1912) sowie seinem Sohn Paul Egon Wildbolz (1905–1994), zusammen mit Georges Major Zur chirurgischen Behandlung der Urogenitaltuberkulose (1956); Peter Brühl (1932–2016) Aktuelle Epidemiologie, Diagnostik und Therapie der Urogenitaltuberkulose DMW 119 S 1121–1125(1994) 10.1055/s-2008-1058812 vgl. Zajakskowiski, T (2012) Genitourinary tuberculosis: historical and basic science review: past and present Cent Europ J Urol.; 65(4) 182–187 10.5173/ceju.2012.04.art1

3 Eine Publikation und Schicksalsklärung zu Hans Haustein ist in Vorbereitung.

4 Im Reichs-Medizinal-Kalender von 1928 ohne Berufssymbol, im Berliner Adressbuch von 1930 Arzt für Haut-Harnleiden und Kosmetik, in der Ausgabe von 1933 des RMK nicht mehr aufgeführt, Entzug der Kassenzulassung 30.06.1933 vgl. Schwoch, R (2009) Berliner jüdische Kassenärzte und ihr Schicksal im Nationalsozialismus. Hentrich, Berlin S 325
